# Unraveling the Synergy between Atezolizumab and Bevacizumab for the Treatment of Hepatocellular Carcinoma

**DOI:** 10.3390/cancers15020348

**Published:** 2023-01-05

**Authors:** Cedric Brackenier, Lisa Kinget, Sarah Cappuyns, Chris Verslype, Benoit Beuselinck, Jeroen Dekervel

**Affiliations:** 1 Department of Gastro-Enterology and Hepatology, University Hospitals Leuven, Herestraat 49, 3000 Leuven, Belgium; 2 Department of General Medical Oncology, Leuven Cancer Institute, University Hospitals Leuven, Herestraat 49, 3000 Leuven, Belgium; 3 Laboratory of Experimental Oncology, Department of Oncology, KU Leuven, Herestraat 49, 3000 Leuven, Belgium; 4 Laboratory of Digestive Oncology, Department of Oncology, KU Leuven, Herestraat 49, 3000 Leuven, Belgium

**Keywords:** immunotherapy, antiangiogenesis, hepatocellular carcinoma

## Abstract

**Simple Summary:**

Hepatocellular carcinoma (HCC) is a highly prevalent cancer with increasing incidence and a high mortality rate. Recently, a combination of an antiangiogenic drug and an immune checkpoint inhibitor, atezolizumab/bevacizumab, has been adopted as a new standard of care to treat advanced HCC. This review discusses the mode of action, clinical efficacy and biomarker research for both drug classes and for the combination therapy with additional insights from the renal cell carcinoma field. Furthermore, a deeper understanding of the pathophysiological mechanisms responsible for the assumed synergy between atezolizumab and bevacizumab is provided.

**Abstract:**

Tyrosine kinase inhibitors (TKIs) with antiangiogenic properties, such as sorafenib, have been the standard choice to systemically treat hepatocellular carcinoma for over a decade. More recently, encouraging results were obtained using immune checkpoint inhibitors, although head-to-head comparisons with sorafenib in phase 3 trials could not demonstrate superiority in terms of overall survival. The IMbrave150 was a breakthrough study that resulted in atezolizumab/bevacizumab, a combination of an antiangiogenic and an immune checkpoint inhibitor, as a new standard of care for advanced HCC. This review discusses the mode of action, clinical efficacy, and biomarker research for both drug classes and for the combination therapy. Moreover, the synergy between atezolizumab and bevacizumab is highlighted, unraveling pathophysiological mechanisms underlying an enhanced anticancer immunity by changing the immunosuppressed to a more immunoreactive tumor microenvironment (TME). This is achieved by upregulation of antigen presentation, upregulation of T-cell proliferation, trafficking and infiltration, impairing recruitment, and proliferation of immunosuppressive cells in the TME. However, more insights are needed to identify biomarkers of response that may improve patient selection and outcome.

## 1. Introduction

The development of systemic treatments for the most common type of primary liver cancer, hepatocellular carcinoma (HCC), has been a long and difficult road. The main reason for this must be sought in the poor activity of systemically administered cytotoxic chemotherapeutics in this cancer type [1,2]. Several resistance mechanisms to chemotherapy have been identified [3]. Additionally, around 80% of HCCs develop in a cirrhotic liver [4]. The impaired liver function in cirrhosis will inevitably affect the patient’s general condition and alter the pharmacokinetics and toxicity profiles of several systemic agents. In this challenging context, options other than cytotoxic agents were explored.

Targeting angiogenesis in a hypervascular solid tumor such as HCC proved to be an attractive strategy. Sorafenib, a tyrosine kinase inhibitor (TKI) with antiangiogenic and antiproliferative properties, was the first approved systemic drug for the treatment of HCC [5]. Positive phase 3 trials investigating drugs with similar profiles followed: lenvatinib [6], regorafenib [7], cabozantinib [8], and ramucirumab [9]. Although the introduction of these drugs resulted in clear progress in the field, their impact remained modest due to limited clinical benefits, primary and acquired resistance, as well as significant toxicities.

This past decade, the added value of immune checkpoint inhibition (ICI) in the treatment of HCC has been thoroughly assessed. Early studies using monotherapy with PD-(L)1 (programmed death-ligand 1) blockade, such as nivolumab [10] and pembrolizumab [11], yielded promising results. Enthusiasm was further enhanced by reports of unprecedented deep and long-lasting remissions in some patients [12]. However, despite a clear signal of activity in a yet-to-define subgroup, subsequent phase 3 registrational trials failed to show improvements in the median overall survival (mOS) versus standard of care in an unselected population [13,14].

Efforts were then directed toward finding drugs that might synergize with anti-PD-(L)1 and improve outcomes in all-comers. The combination of atezolizumab (anti-PD-L1) and bevacizumab (anti-VEGF (vascular endothelial growth factor)) was the first regimen to show an improvement in overall survival (OS) versus sorafenib in the first-line systemic treatment of HCC [15], establishing a new standard of care. Recent data from the HIMALAYA trial also showed superiority for the combination of durvalumab (anti-PD-L1) and tremelimumab (anti-CTLA (cytotoxic T-lymphocyte-associated protein)-4) versus sorafenib in the first-line treatment of HCC, establishing an alternative option for atezolizumab/bevacizumab [16].

The development of several efficacious drugs in the field of HCC has created multiple treatment options in various possible sequences [17]. In order to further finetune treatment decisions as well as develop new strategies for patients that are currently poorly served, a detailed understanding of the working mechanism of the available agents is necessary. Moreover, validated biomarkers for responses are needed to facilitate treatment decisions. Among different cancer types, renal cell carcinoma (RCC) resembles HCC the most, both in terms of its tumor biology and in its challenges to develop effective treatments with clinically usable predictive biomarkers. Similar to HCC, the tumor microenvironment (TME) of RCC is characterized by increased angiogenesis, which in RCC is due to the loss of function of the *Von-Hippel Lindau* gene, and a high immune infiltration largely dominated by immunosuppressive features resulting in an immunosilenced TME [18,19]. These characteristics explain why drug development runs a similar and largely parallel course. Both in HCC and RCC chemotherapy is ineffective, antiangiogenic-targeted treatment revolutionized the treatment landscape and combination therapies of either anti-VEGF/ICI or anti-PD-(L)1/CTLA-4 inhibitors have currently become the standard of care for the first-line treatment [20]. 

This review provides a summary of the current knowledge on both drug classes that are part of the atezolizumab/bevacizumab combination as well as areas of possible synergy. Translational and (pre-)clinical data in the field of HCC is supplemented by findings in the field of RCC in order to provide maximal insight into the reasons for the success and failure of the current standard of care.

## 2. The Current Position of Systemic Agents in the Treatment of HCC

In recent years, together with the emergence of new agents to treat HCC, the position of systemic treatment in the treatment algorithm has changed [21,22].

The Barcelona Clinic Liver Cancer (BCLC) staging system links tumor stage, liver function, cancer-related symptoms, and performance state (PS) to an evidence-based treatment algorithm that guides clinicians in treatment decision-making. It uses five stages: very early stage (0), early stage (A), intermediate stage (B), advanced stage (C), and terminal stage (D). BCLC stages 0, A, and B all include ECOG (Eastern Cooperative Oncology Group) PS 0 and preserved liver function; with BCLC-0 existing of one single lesion ≤2 cm, BCLC-A existing of one single lesion >2 cm or ≤3 lesions each ≤3 cm and BCLC-B containing the multinodular disease. BCLC stage C is defined as HCC with portal invasion, extrahepatic spread, and/or patients with ECOG PS 1 or 2, all with preserved liver function. BCLC stage D is defined as patients with end-stage liver function and ECOG PS 3 or 4 due to the tumor burden [17].

The current BCLC guideline positions systemic therapy as the first-line treatment in BCLC stage C, but also as the first treatment line in a subgroup of BCLC stage B and as an option for downstaging [17]. This is in contrast with previous guidelines where systemic therapy was only indicated as the first-line treatment option in the BCLC-C stage or as the second-line treatment in the BCLC-B stage with contraindications or with untreatable progression after transarterial chemoembolization (TACE) [23].

The 2022 BCLC version stratifies the BCLC-B stage into three groups according to the tumor burden and liver function. The first subgroup reaching extended liver transplantation (LTX) criteria can benefit from LTX. The second subgroup consisting well-defined nodules, preserved portal flow, and selective access can benefit from TACE. The third subgroup consisting of diffuse, infiltrative, extensive, and bilobar liver involvement are now candidates for systemic therapy as the first-line treatment. Moreover, patients with BCLC-B, with well-defined lesions but neither fulfilling extended LTX criteria nor TACE criteria must be considered for systemic therapy. It is difficult to determine strict evidence-based criteria to recommend systemic treatment over TACE, underlining the role of expert assessment and the need for deeper investigation into the position of systemic therapy within these subgroups. Phase 3 studies evaluating the role of systemic therapy as combination therapy with TACE, in the adjuvant setting after TACE or instead of TACE are pending [17,24].

Systemic therapy can also be used to downstage tumor burden. Within BCLC-A, a single lesion that cannot be resected and is beyond the size for effective TACE, systemic therapy may be helpful to reduce the size and enable subsequent local therapy. Even cases of advanced HCC (aHCC) treated with immunotherapy and followed by LTX after spectacular response have been published [25]. However, more evidence is needed to define the role of systemic therapy as a neoadjuvant treatment before LTX and its use is not yet integrated into the updated guidelines [25].

## 3. Antiangiogenics in the Treatment of HCC

### 3.1. Mechanism of Action of Antiangiogenics

Angiogenesis refers to the formation of new blood vessels, a complex process that is mediated by pro- and antiangiogenic factors. Proangiogenic factors have been studied extensively. The most important include the VEGF family, angiopoietins, epidermal growth factors, and basic fibroblast growth factors. On the other hand, antiangiogenic factors such as TSP and angiostatin are less well characterized [26,27]. Furthermore, it is well-established that inflammatory molecules such as IL-6 and IL-8 also play a role in angiogenesis. Angiogenesis is initiated by the destabilization of existing microvasculature, which leads to vascular hyperpermeability, remodeling of the extracellular matrix, and endothelial cell activation [28,29]. 

Malignancies hijack and dysregulate the physiological mechanisms of angiogenesis. Tumoral neo-vascularization is promoted by an excess of proangiogenic factors, such as VEGF, which are secreted by tumor and stromal cells in response to several stimuli of which hypoxia is the most important. These novel blood vessels are of remarkably low quality, characterized by chaotic organization, immaturity, and leakiness [30].

The general principles of antiangiogenic treatment have been thoroughly reviewed in the past [31,32]. The initial aim of blocking angiogenesis was to cut off blood supply to the tumor, resulting in increasingly severe hypoxia, deprivation of critical nutrients, and eventually necrosis of tumoral lesions. An alternative or coexistent mechanism has since then been suggested [33]. In a certain small therapeutic window, antiangiogenics are thought to contribute to vascular normalization, which entails the restoration of proper blood vessels in the tumor, facilitating the perfusion of nutrients and oxygen but also of therapeutic agents and infiltrating immune cells. While supplying oxygen to cancer cells could be regarded as an undesired effect, it is well known that cells surviving hypoxia switch to a more aggressive, invasive phenotype [34]. Currently approved antiangiogenics in the field of HCC include antibodies that block the VEGF-ligand (bevacizumab), the VEGF-receptor 2 (ramucirumab) as well as multi-target TKIs that block the kinase activity of the VEGF-receptors (sorafenib, lenvatinib, regorafenib, and cabozantinib). Of note, the latter group inhibits many other kinases, such as c-kit, FGFR, MET, and RET [35]. For the purpose of this review, we will focus on their antiangiogenic properties.

### 3.2. Clinical Efficacy of Antiangiogenics in HCC

Five drugs with antiangiogenic properties have shown survival benefits in phase 3 clinical trials and have been approved for frontline or later-line treatment of patients with aHCC: sorafenib [5], lenvatinib [9], regorafenib [6], ramucirumab [7], and cabozantinib [8].

The first positive trial investigating a systemic treatment for aHCC dates from 2008. The breakthrough phase 3 SHARP trial compared sorafenib to placebo as the first-line treatment in aHCC and demonstrated an OS benefit from 7.9 to 10.7 months (hazard ratio(HR) [95% confidence interval (CI)]: 0.69 [0.55–0.87], *p* < 0.001). Treatment with sorafenib also led to significantly better disease control (43% vs. 32%, *p* = 0.002) and a longer time to radiological progression (5.5 vs. 2.8 months; HR [95% CI]: 0.58 [0.45–0.74], *p* < 0.001) [5]. In the REFLECT trial, lenvatinib was shown to be non-inferior to sorafenib in the first-line treatment with a mOS of 13.6 months vs. 12.3 months, respectively (HR [95% CI]: 0.92 [0.79–1.06]). Lenvatinib also demonstrated significant improvement for all secondary endpoints including progression-free survival (PFS) (7.3 vs. 3.6 months; HR [95%CI]: 0.64 [0.55–0.75] *p* < 0.001), and an objective response rate (ORR) (40.6% vs. 12.4%; OR [95%CI]: 3.34 [2.17–5.15], *p* < 0.001) [6].

Regorafenib was the first approved second-line therapy (after progression on sorafenib) for aHCC based on the findings of the RESORCE trial. An improvement in OS was demonstrated with mOS of 10.6 months in the regorafenib-arm compared to 7.8 months with the placebo (HR [95%CI]: 0.63 [0.50–0.79], *p* < 0.001) [7]. Ramucirumab improved mOS in patients who progressed or were intolerant to sorafenib, but only for patients with baseline AFP (alpha-fetoprotein) ≥400 ng/mL (REACH-2 trial) with mOS of 8.5 months for ramucirumab vs. 7.3 months with placebo (HR [95%CI]: 0.71 [0.53–0.95], *p* = 0.020) [9]. Finally, in the CELESTIAL trial, cabozantinib demonstrated an improved mOS compared to the placebo for the second and third line in aHCC (with sorafenib as one of the previous lines): 10.2 vs. 8.0 months (HR [95%CI]: 0.76 [0.63–0.92], *p* = 0.005) [8].

There are no randomized controlled trials investigating bevacizumab monotherapy in aHCC. Nevertheless, monotherapy with bevacizumab has been investigated in eight phase 2 trials reporting mOS ranging from 4.4 to 15.7 months. Interestingly, six of the eight trials reported mOS rates that were similar to or greater than those in the SHARP trial [36].

Unfortunately, the clinical benefit of antiangiogenic agents in HCC is modest. This is partly due to their challenging toxicity profile. TKIs are particularly difficult, causing hand-foot-syndrome, anorexia, diarrhea, and hypertension, while proteinuria and hypertension (both with risk of impairment of renal function) are the most common toxicities for antiangiogenic monoclonal antibodies [37]. As a result, dose reductions or treatment interruptions are frequent (e.g., 64% of patients in the SHARP trial) [5]. Additionally, the presence of primary resistance to antiangiogenics in many patients and the emergence of acquired resistance are important hurdles that can have a dramatic impact on the course of the disease. Reliable biomarkers of response for patient selection, as well as combination treatments to overcome resistance, are two main strategies to tackle these issues [38]. 

### 3.3. Potential Biomarkers for Antiangiogenics in HCC

Predictive biomarkers will enable the identification of a subgroup of patients that will benefit from a specific therapy. In contrast, prognostic biomarkers are merely informative in foreseeing the outcome in general, unrelated to a treatment [39]. Most studies focus on blood and tissue markers in response to sorafenib, as it was the only approved first-line treatment for aHCC for a long time. However, in the last decade, the research field of biomarkers has broadened enormously. Related to the molecular heterogeneity of HCC and multiple pathways of angiogenesis, finding reliable predictive biomarkers for this drug class has proved to be particularly challenging. Furthermore, in aHCC specifically, tumor biopsies are not always available due to the frail patient population and the fact that biopsies are not mandatory for diagnosis, which hampers tissue marker research. In Table 1, an overview of candidate biomarkers in HCC and RCC is given.

To date, **AFP** is the only validated predictive biomarker in HCC, in particular, high AFP levels (>400 ng/mL) are predictive for the response to ramucirumab in the second-line treatment [17,40]. The REACH trial investigated ramucirumab as a second-line treatment in patients with aHCC after first-line sorafenib but could not reach its primary endpoint of OS. Nonetheless, a subgroup analysis suggested an increase in OS in patients with high AFP. This led to a new phase 3 trial named REACH-2: the first biomarker-enriched phase 3 trial in HCC where patients with a level of AFP more than 400 ng/mL were enrolled and treated with ramucirumab in the second line after sorafenib. This study demonstrated an increase in OS of 1.2 months compared to the placebo (HR [95% CI]: 0.694 [0.571–0.842]) [9]. The underlying mechanisms of overexpression of AFP and the biological characteristics of patients with high levels of AFP are not fully understood. Analyzing the molecular profile of HCC patients, an inverse correlation between AFP promoter methylation and AFP expression was seen. AFP-high tumors were characterized by poor differentiation, enrichment of progenitor features, and enhanced proliferation [41]. Tumors expressing AFP are also associated with more stem cell-like features (such as EpCAM expression) and increased VEGF pathway activity [42]. Furthermore, gene expression profiling found that activation of Wnt/β-catenin signaling, which is known to promote angiogenesis, was mainly associated with the AFP+/EpCAM+-subgroup [43]. The increased EpCAM-expression and Wnt/β-catenin signaling leading to increased VEGF pathway activity are possible explanations for the predictive role of high AFP for the response to ramucirumab. However, these hypotheses do not explain why AFP has no predictive value for the treatment with antiangiogenic TKIs, such as sorafenib [44,45]. Besides its predictive role, AFP has a clear prognostic value and is adopted in scoring scales, used as inclusion or exclusion criteria in studies, and guidance in the selection for LTX [17]. 

A wide array of potential circulatory biomarkers in HCC have been researched, but none have been validated as predictive markers. VEGF-A and angiopoietin-2 (Ang2) do have the strongest relationship with the outcome [46]. As VEGF is one of the key regulators in angiogenesis, **soluble VEGF-A** (the most important member of the VEGF family for angiogenesis) seemed to be a promising biomarker. In the SHARP trial, the mOS of patients with low and high baseline VEGF concentrations was 10.6 and 6.2 months respectively (HR [95% CI]: 1.53 [1.19–1.96], *p* < 0.001) confirming its prognostic value. However, as it was not associated with a higher disease control rate or longer PFS, it was not validated as a predictive marker for sorafenib [46] or bevacizumab [38,47]. **Ang2** is an important proangiogenic factor that, in the presence of VEGF, destabilizes blood vessels and enhances vascular sprouting. High plasma Ang2 concentrations at baseline are indicative of poor prognosis in patients with aHCC, suggesting that elevated levels of this angiogenic factor may be associated with more aggressive disease. In the SHARP trial, the mOS rates of patients with low and high baseline Ang2 concentrations were 14.1 and 6.3 months, respectively (HR [95% CI]: 2.40 [1.91–3.03], *p* < 0.001). In accordance with VEGF-A, Ang2 has a proven prognostic, but no predictive value [46,48,49].

As HCC shares key tumor biology hallmarks with RCC and angiogenesis inhibitors are a cornerstone of both cancer treatment options, a comparison with the current RCC biomarker research field can, therefore, provide valuable insights to guide future research for HCC biomarkers.

Prognostic classifications based on clinical and biochemical criteria have been developed for RCC, notably the International Metastatic RCC Database Consortium (IMDC) risk model. Favorable-risk patients have better outcomes to VEGFR-TKIs, likely reflecting a more angiogenic and less inflamed TME, and therefore better response to angiogenesis inhibitors [50].

Similarly to HCC, baseline VEGF levels have been studied as potential circulatory biomarkers. Lower VEGF levels correlate with improved outcomes with VEGFR-TKIs, but this is a prognostic rather than predictive effect and, therefore, has little use for treatment selection [51,52,53]. Single nucleotide polymorphisms (SNP) in components of angiogenesis pathways, in particular VEGFR1, have been shown to impact response on angiogenesis inhibitors [54,55]. In particular rs7993418, a synonymous SNP affecting the VEGFR1 tyrosine-kinase domain, correlated significantly with PFS in the bevacizumab group in the AVOREN trial and could therefore serve as a selection marker for treatment with bevacizumab in RCC [56]. However, the clinical application remains challenging due to the lack of validation and low prevalence of specific SNPs, which also vary across populations of different ethnicities [57]. 

To date, transcriptomic biomarkers are the most promising for future clinical applications. Gene signatures reflecting heightened angiogenesis have been correlated with improved outcomes on VEGFR-TKIs in the first line. This was demonstrated in several retrospective studies, but also in biomarker analyses of large phase 3 trials comparing IO-VEGF targeting therapies with VEGFR-TKI monotherapy [58,59,60]. Through analysis of transcriptomic profiles, distinct molecular subtypes have been described within clear-cell RCC. Subgroups characterized by higher angiogenesis are correlated with improved outcomes to VEGFR-TKIs, whereas more inflamed subtypes generally respond poorly [61,62]. RCC tumors with hyper-angiogenic subtypes are overrepresented in the IMDC favorable risk groups and have more indolent tumor behavior. Additionally, these tumors are more likely to develop metastases within glandular organs, a clinical feature with a known association of indolent tumor behavior [63,64].

Notably, results of the first biomarker-driven randomized phase 2 trial in metastatic RCC using molecular subtypes were recently published. In the angiogenesis-high ccRCC (clear-cell renal cell carcinoma)2 subgroup, patients were randomized between sunitinib monotherapy and combination ICI therapy. Both groups had comparable results indicating that for patients harboring a tumor with ccrcc2 phenotype, in particular those with favorable risk characteristics and low expressions of immune-related signatures, monotherapy sunitinib may be non-inferior to dual ICI in the first line [65].

These promising results require further prospective validation before they can be incorporated into treatment guidelines and transcriptomic biomarkers pose technical challenges for routine use in clinical practice. Nevertheless, an improved understanding of tumor behavior and disease course of the various subtypes may aid in treatment decisions in absence of clinically implemented biomarkers [66,67]. Specifically for aHCC, tissue for transcriptomic biomarker development and validation is scarce. 

## 4. Immune Checkpoint Inhibition in the Treatment of HCC

### 4.1. Mechanism of Action of Immune Checkpoint Inhibitors

Immune-checkpoint molecules are key elements of the adaptive immune response that modulate the activation status of the immune system. PD-1 and CTLA-4 are two of the main inhibitory immune-checkpoint molecules that temper the activation of the immune system in order to avoid deleterious overstimulation. The inhibitory interaction between PD-1 and its ligand PD-L1 leads to T-cell inactivation, while increased CTLA-4 expression on T-cells inhibits effective antigen presentation in the immunological synapse and reinforces the immunosuppressive function of regulatory T-cells (Tregs; a subpopulation of T-cells that have immunosuppressive properties thereby maintaining homeostasis and self-tolerance). It is well-established that cancer cells are capable of hijacking these built-in defense mechanisms, dampening the anti-tumor immune response, thereby avoiding elimination and enhancing further tumor progression. 

Therapeutically targeting these immune-checkpoint molecules has revolutionized anti-cancer immunotherapy and has led to clinically meaningful benefits in a wide array of solid tumors, including HCC, albeit for a subset of patients [68,69,70,71,72]. Checkpoint inhibitors that target the PD-1/PD-L1 axis and CTLA-4 are the most widely used, and arguably, the most successful forms of anti-cancer immunotherapy. Therapeutic targeting of CTLA-4 releases the CTLA-4-dependent ‘brake’ on the immune system leading to more effective priming of T-cells by cancer antigens and restoring the balance between stimulatory and regulatory signals in the TME [73]. In contrast, anti-PD-(L)1 immunotherapy breaks the inhibitory interaction between PD-1 and PD-L1, thereby re-invigorating tumor-specific cytotoxic T-cells (CTLs; a subpopulation of T-cells that can directly destroy cells after a specific antigen presentation). Furthermore, as new antigens are released through apoptosis of cancer cells, checkpoint inhibitors play an important role in maintaining anti-tumor immunity by recruiting, priming, and activating novel tumor-specific T-cells clones, so-called clonal replacement [74].

The liver is a frontline immunologic organ and an important first line of defense against the influx of pathogens from the gastrointestinal tract that reach the liver via the enterohepatic pathway [75]. Consequently, the healthy liver is highly populated with both innate and adaptive immune cells that create an overall immunotolerant microenvironment. However, in the context of chronic inflammation, the composition of immune cells in the liver can change dramatically, disturbing the fine balance between stimulatory and inhibitory signals and ultimately leading to progressive liver damage and eventually malignant transformation and the development of HCC [76,77]. 

An altered microenvironment is key in the pathogenesis and prognosis of HCC. The distinct viral and non-viral etiologies are associated with a unique TME. The immune traits of the TME HCCs can be characterized as immune active tumors, which tend to respond well to ICI, or immune-excluded tumors which are proposed to be resistant to ICI. Viral hepatitis-driven HCC has typically an exhausted immune TME whereas (N)ASH-induced HCC is characterized by an activated immune TME [22,72]. However, it is clear that the immune context of liver cancer is distinct from other cancer types. For example, unlike most cancer types, the presence of PD-1+ CD8+ T-cells (CD8+ T-cells express CD8 on their surface and evolve toward CTLs after priming) has been linked to a worse prognosis in HCC [78]. In fact, in pre-clinical NASH (non-alcoholic steatohepatitis) HCC models, the progressive accumulation of unconventionally activated–dysfunctional PD-1+ CD8+ T-cells was involved in impaired tumor surveillance and actually led to further tumor progression [79]. 

### 4.2. Clinical Efficacy of Immune Checkpoint Inhibitors in HCC

Though checkpoint inhibitors have revolutionized the treatment of several solid tumors, including aHCC, only around 15–20% of HCC patients exhibit an objective response to ICI monotherapy [80].

#### 4.2.1. PD-1/PD-L1 Inhibitors

Nivolumab (anti-PD-1) granted FDA approval for aHCC after sorafenib failure based on results of phase 1/2 trial CheckMate 040 with ORR of 15% (95% CI 6–28) and OS of 15.1 months (95% CI 9.6–20.2) [10]. Despite these encouraging results, OS for nivolumab was not significantly longer than for sorafenib in the first-line treatment as observed in the phase 3 trial Checkmate 459: 16.4 months (95% CI 13.9–18.4) for nivolumab versus 14.7 months (95% CI 11.9–17.2) for sorafenib with HR 0.85; *p* = 0.075 [13].

Pembrolizumab (anti-PD-1) was also granted FDA approval for aHCC after sorafenib based on results of the phase 2 trial KEYNOTE-224 with ORR 16.3% (95% CI 9.8–24.9) with OS 12.9 months (95% CI 9.7–15.5) [11]. Moreover, these promising results were not confirmed in the phase 3 KEYNOTE-240 trial comparing pembrolizumab to placebo as the second-line treatment for aHCC after the failure of sorafenib. Median OS was 13.9 months (95% CI 11.6–16.0) for pembrolizumab versus 10.6 months (95% CI 8.3–13.5) for placebo with HR 0.78 (95% CI 0.611–0.998); *p* = 0.024 but this did not meet the prespecified boundary of significance [14]. A recently reported study in Asia, KEYNOTE-394, reported a significantly improved OS, PFS, and ORR in patients treated with pembrolizumab compared to the placebo in patients with aHCC previously treated with sorafenib [81]. In the recently presented open-label, multicenter, randomized phase 3 HIMALAYA trial patients with advanced HCC without prior systemic therapy were randomized into three different treatment arms: tremelimumab (anti-CTLA-4) plus durvalumab (anti-PD-L1), durvalumab monotherapy, or sorafenib. An important difference between patient selection between IMbrave150 and HIMALAYA is the presence of portal vein thrombosis (exclusion criterium in HIMALAYA in contrast to IMbrave150). Durvalumab (anti-PD-L1) demonstrated non-inferior OS versus sorafenib with OS 16.6 months (95% CI 14.1–19.1) for durvalumab versus 13.8 months (95% CI 12.3–16.1) for sorafenib [16]. A recent press release announced that the anti-PD-1 antibody tislelizumab was found non-inferior to sorafenib in terms of OS. More detailed results are pending [82].

#### 4.2.2. CTLA-4 Inhibitors

Tremelimumab has been tested as monotherapy in aHCC with chronic hepatitis C. Partial response rate was 17.6% and the time to progression was 6.5 months (95% CI 4.0–9.1) [83]. Nowadays, most CTLA-4 inhibitors are used in combination therapies for aHCC treatment (e.g., ipilimumab–nivolumab). No CTLA-4 inhibitor is approved for HCC treatment as monotherapy [84].

#### 4.2.3. Other Immune Checkpoint Inhibitors

Beyond PD1, PDL1, and CTLA4, other immune checkpoint inhibitors were developed and are under study in HCC. The expression of TIM3 negatively regulates the T cell effector function and enhances the suppressor function of Treg. Different studies of anti-TIM3 as a (co)therapy in HCC are recruiting (e.g., NCT03680508) [85]. LAG3 binds MHC class II molecules and has an inhibitory function to CTLs. Recently a benefit of combining a LAG3 inhibitor to anti-PD1 therapy has been demonstrated in a randomized controlled trial (RCT) in melanoma and the first antibody against LAG3 has been FDA-approved for melanoma [86]. No clinical data of ICI other than PD1, PDL1, or CTLA4 in phase 3 RCT in HCC are available at this moment.

#### 4.2.4. ICI-Combination Therapies

In the HIMALAYA trial, a combination of durvalumab (anti-PD-L1) and tremelimumab (anti-CTLA-4) led to a significant improvement in OS versus sorafenib [16.4 months (95% CI 14.2–19.6) for durvalumab/tremelimumab versus 13.8 months (95% CI 12.3–16.1) for sorafenib], meeting the primary endpoint of the study (HR 0.78; 96% CI 0.65–0.92; *p* = 0.004). The ORR was 20% with durvalumab/tremelimumab versus 5% with sorafenib. The amount of major adverse effects was similar in both groups. The results of the HIMALAYA trial provide durvalumab/tremelimumab as an alternative to atezolizumab/bevacizumab [16]. The combination of nivolumab (anti-PD-1) and ipilimumab (anti-CTLA-4) was evaluated in the phase 1/2 trial Checkmate 040 as the second-line treatment after sorafenib. Median OS was 22.8 months (95% CI 9.4-not reached) in the arm with the highest dose of the combination therapy. The toxicity profile of that treatment arm was high with adverse effects of any grade of 94%. As this combination therapy gave the longest OS seen in the second-line treatment after sorafenib, it led to an accelerated FDA approval [10,87].

### 4.3. Potential Biomarkers for Immune Checkpoint Inhibitors in HCC

As HCC patients are often fragile and have only a narrow window of opportunity for treatment, biomarker-driven treatment solutions are highly needed. However, many efforts and promising (theoretical) concept results of research in biomarkers for HCC are mostly disappointing with currently no predictive biomarker for ICI response available in clinical practice. The most researched potential biomarkers are PD-L1 expression in tumor cells and/or immune cells, tumor mutational burden, and mismatch repair deficiency and activation of the Wnt/β-catenin pathway. The immune signature, T-cell receptor (TCR) analyzation, antidrug antibodies, and even gut microbiome could serve as additional values [88]. Considering the complexity of the response to checkpoint inhibitors, presumably, a predictive model incorporating several factors (genetics and environmental) will be more likely to estimate the probability of response to ICI, rather than a single biomarker.

**Expression of PD-L1** is associated with tumor aggressiveness and poor prognosis [89]. Despite the proven association of PD-L1 on immunohistochemistry and response to ICI and its approved diagnostic assay as the biomarker for the treatment of several cancers (e.g., non-small-cell lung, urothelial and gastric cancers) it is not a validated tool for ICI response in HCC. Tumor proportion score, which is the percentage of viable tumor cells with partial or complete membrane staining of PD-L1 relative to all viable tumor cells present in the sample (positive if ≥1%), was not predictive for the response to pembrolizumab in KEYNOTE-224 [10]. On the other hand, the combined positive score, which is the percentage of PD-L1-positive cells (besides tumor cells also stromal immune cells) over the total number of viable tumor cells, was associated with a response to pembrolizumab and improved PFS [10,11]. In the Checkmate 459 study, the proportion of responders in the nivolumab group was also higher, although not significantly higher, in patients with tumor cell PD-L1 expression of 1% and greater compared with patients with PD-L1 expression less than 1%. Tumor cell PD-L1 expression was not predictive for OS or PFS [13]. The heterogeneity of PD-L1-testing assays and relatively small sample sizes might contribute to the inconsistency of the observed results [90]. Awaiting analyses of PD-L1 expression and its association with survival endpoints from pending and future RCTs of ICIs in monotherapy as well as in combination regimens will strengthen our understanding and hopefully enhance the feasibility of this tool as a clinically useful biomarker.

**Activated Wnt/β-catenin signaling** has been associated with immune exclusion and, consequently, immunotherapy resistance in HCC and other cancer types, such as melanoma. Mutations in CTNNB1, which encodes β-catenin, occur in one-third of HCC patients. In CTNNB1-mutant murine and human tumor samples a reduction in CCL5, a chemokine dealing with the recruitment of CD103+ dendritic and antigen-specific CD8+ T cells, was observed. As a consequence, activated Wnt/β-catenin signaling leads to the impaired recruitment of dendritic cells, which are critical for establishing an effective anti-tumor immune response by processing antigen material, presenting it on their surface and the activating T-cells. Moreover, it is probable that the level of β-catenin activation is dependent on mutations co-occurring with CTNNB1-mutation. Additional studies are needed to refine the set of mutations that cooperate with CTNNB1 mutation to confer ICI resistance. Therapeutic options that promote dendritic cell recruitment could improve the response of CTNNB1-mutant tumors to ICI [80,88].

High **tumor mutational burden** (TMB), defined as the number of nonsynonymous single nucleotide variants, may increase the likelihood of ICI response. However, in HCC, TMB is generally low and its role as a predictive marker for ICI is not supported by available data [91,92]. 

Response to ICI in the first-line treatment in patients with aHCC was associated with upregulation in interferon-y (IFN-y) signaling and gene sets related to antigen presentation machinery. An 11-**gene signature** related to the IFN-y and antigen presentation machinery was found to be significantly associated with predicting ORR, PFS, and OS in patients with aHCC receiving ICI in the frontline. However, in patients who were treated with TKIs in the frontline before receiving subsequent ICI, the signature was no longer able to predict either OS, PFS, or ORR. This signature was also validated for ICI response in other cancer types. In HCC, high expression of this signature was associated with a distinct profile in the immune infiltrate, marking an increase in M1 macrophages (macrophages (innate immune cells capable of phagocytosis and destruction of antigens) involved in pro-inflammatory responses), CD4+ memory T-cells (a subset of so-called T-helper cells with an antigen-specific memory that remains long-term after antigen disappearance creating an augmented immune response after reactivation) and CD4+ naïve T-cells (a subset of so-called T-helper cells that has not been activated through an encounter with their target antigen) [93]. These gene signatures need further evaluation.

A recent study evaluated the crosstalk between the tumor and peripheral immune system using single-cell RNA-sequencing and single-cell TCR-sequencing of both tumor and peripheral blood samples of aHCC patients treated with ICI. Interestingly, the composition of tumor-infiltrating T-cells was similar in ICI-responding and non-ICI-responding tumors before treatment. Analyses of tumor tissue showed that ICI-responding tumors were characterized by a significantly higher TCR-clonality pretreatment versus non-ICI-responding tumors that were characterized by a more diverse, non-clonal TCR-repertoire. Moreover, by combining TCR-sequencing in the tumor and blood, it was observed that ICI-responding tumors had a higher degree of TCR-sharing (between tumor and peripheral blood). The concept of **TCR-clonality and TCR-sharing between tumor and peripheral blood** are potential candidates for predicting ICI response but further research is needed before use in clinical practice. Furthermore, CXCL10 positive macrophages were the most important PD-L1 expressing cell type in aHCC with the level of expression correlating with the response [94].

Other recent studies using single-cell analyses on tumor and peripheral immune cells identified CXCR3 + CD8+ effector memory T-cells in blood and CD103+ tissue-resident memory T-cells in the tumor associated with the response to ICI in HCC [78,95]. 

In RCC, prognostic risk categories were developed using clinical and biochemical criteria. Intermediate and poor risk diseases are enriched in subtypes high in proliferation and inflammation, which generally respond worse to VEGFR-TKIs; whereas favorable-risk patients have a higher proportion of angiogenesis-high tumors, responding better to VEGFR-TKIs [50]. This might explain why in the phase 3 trial comparing ipilimumab–nivolumab to sunitinib, no OS benefit was seen in the favorable risk group, which led to the approval of this combination only in patients with intermediate or poor risk [96]. Other circulatory biomarkers include a neutrophil-to-lymphocyte ratio (NLR), defined as neutrophil count divided by lymphocyte count. A higher NLR reflects pro-tumorigenic systemic inflammation and correlates with a worse prognosis across cancer types [97]. Moreover, an increase of NLR during the first six weeks of anti-PD-1 treatment in RCC is associated with worse PFS and OS, and could potentially serve as an early indicator of treatment resistance [98,99].

PD-L1 positivity is associated with longer PFS in patients receiving a combination of atezolizumab/bevacizumab versus sunitinib, but it is not associated with OS [100]. A recent meta-analysis demonstrated that PD-L1 positivity is correlated with improved ORR and PFS across different ICI-containing treatment regimens in RCC, nevertheless in several phase 3 trials, it failed to demonstrate an impact on OS [101,102,103,104]. Therefore, as with HCC, the PD-L1 status is currently not recommended as a clinical biomarker in RCC. TMB is another intensely investigated potential biomarker that has failed to demonstrate an impact on the response to ICI in phase 3 trials. Similar to HCC, RCC also has generally low TMB [60,102].

Currently, molecular biomarkers hold the most promise for application in clinical practice. Transcriptomic immune-related gene signatures have been developed in biomarker analyses of two RCTs in RCC. Signatures reflecting T-effector activation and general immune response have been correlated with the response to ICI, whereas increased myeloid infiltration may predict resistance to ICI monotherapy [60].

Furthermore, RCC molecular subtypes derived from transcriptomic profiling have differing sensitivity to ICI. Analysis of the IMmotion151 cohort showed that subgroups characterized by T-effector and/or cell cycle signaling had superior outcomes on atezolizumab/bevacizumab compared to VEGFR-TKI monotherapy. In a recent first-in-class biomarker-driven trial, a combination of ipilimumab–nivolumab achieved better responses than nivolumab monotherapy in immune-cold ccrcc1 tumors, whereas both treatments gave comparable results in the immune-inflamed ccrcc4 tumors. These findings suggest that for a subgroup of patients, ipilimumab and its added toxicity could be omitted without compromising outcomes [65]. Further prospective validation of these subgroups is ongoing.

A recent biomarker analysis has highlighted the biomarker potential of TCR-repertoire in RCC as well. TCR-sequencing from bulk tissues revealed that the existence of pre-treatment expanded TCR-clones, and in particular the maintenance of these clones, correlated to anti-PD-1 response [105]. This is in line with recent research data in HCC.

## 5. Atezolizumab with Bevacizumab for the Treatment of HCC

### 5.1. Mechanism of Action of the Combination

The combination of PD-L1-inhibition and VEGF-inhibition has dramatically improved the efficacy of systemic treatment in aHCC. The reason for the success of combination therapy must be sought in the immunosuppressed state of the TME of HCC and the capability of (anti)angiogenesis to modulate this. 

**Figure 1:**
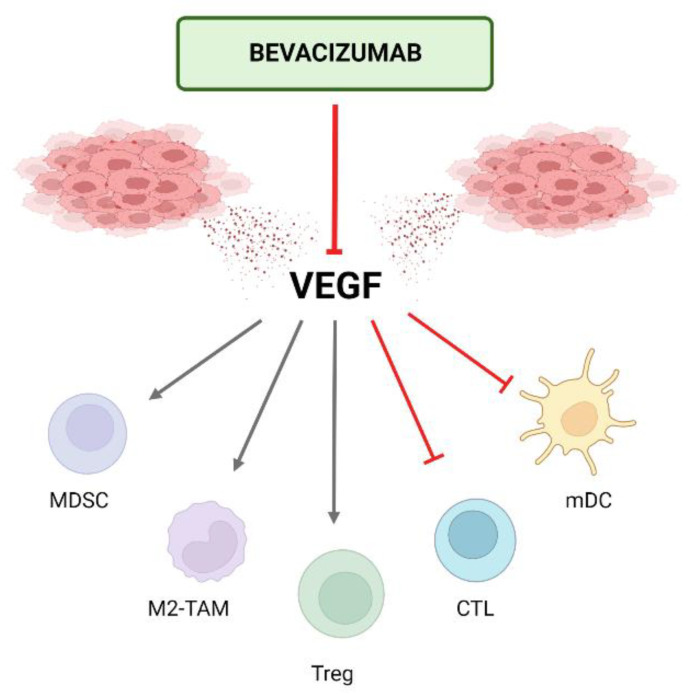
Direct effects of VEGF on different types of immune cells. VEGF, which is secreted by tumor and stromal cells, directly increases the proliferation of MDSCs, Tregs, and M2-like TAMs that have immunosuppressive functions and inhibit the proliferation or maturation of CTLs and mDCs that have immunoactivating functions. As a result, VEGF creates an immunosilenced tumor microenvironment. Bevacizumab can counteract these actions and, therefore, turn the immunosuppressed toward an immunoactive tumor microenvironment. VEGF: vascular endothelial growth factor; MDSC: myeloid-derived suppressor cell; Treg: regulatory T-cell; TAM: tumor-associated macrophage; CTL: cytotoxic T-cell; mDC: mature dendritic cell. Created with Biorender.com; no copyright issue.

**Figure 2:**
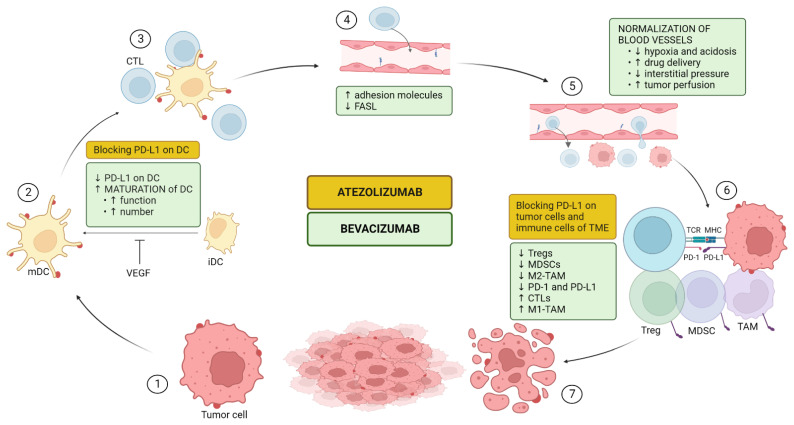
Effects of atezolizumab and bevacizumab upon the cancer-immunity cycle (CIC): enhancing anticancer immunity by creating a more immunoactive environment. Antigen presentation via DCs is upregulated due to bevacizumab-induced maturation of DCs and to blocking or decreasing expression of PD-L1 on DCs. As a result, more CTLs are activated. Bevacizumab facilitates the infiltration of these activated CTLs into the blood vessel by increasing adhesion molecules and by ‘vascular normalization’. This latter generally means a less chaotic organized and leaky endothelium leading to a less hypoxic and acidic tumor microenvironment (TME), less interstitial pressure, and more drug delivery and tumor perfusion. Furthermore, bevacizumab decreases Fasl, an antigen that can induce apoptosis of CTLs. In the tumor, the activated CTLs will evade the vessel and kill cancer cells in the presence of an immunofavorable TME. Bevacizumab will enhance that environment by, directly and indirectly, increasing immunoactivating cells (CTLs and M1-TAMs) and decreasing immunosuppressor cells (Tregs, MDSCs, and M2-TAMs). Finally, atezolizumab and bevacizumab will both respectively block or decrease PD-(L)1 on tumor cells and the immune cells of the TME. Both therapies play a role in augmenting cancer antigen presentation, activation of T-cells, and recognition of cancer cells by CTLs; bevacizumab additionally augments trafficking and infiltration of T-cells to the tumor. Taken together, these therapies synergistically create a more immunoreactive TME. Steps of the CIC: 1. Release of cancer antigens; 2. Cancer antigen presentation; 3. Priming and activation of T-cells; 4. Trafficking of T-cells to the tumor; 5. Infiltration of T-cells into the tumor; 6. Recognition of cancer cells by cytotoxic T-cells (CTLs); 7. Lysis of cancer cells. DC: dendritic cell; mDC: mature dendritic cell; iDC: immature dendritic cell; VEGF: vascular endothelial growth factor; FASL: Fas ligand; Treg: regulatory T-cell; MDSC: myeloid-derived suppressor cell; TAM: tumor-associated macrophage. Created with BioRender.com; no copyright issue.

In addition to its well-characterized role in angiogenesis, VEGF also drives immunosuppression in the TME either directly or indirectly via three principal mechanisms as described below. In Figure 1, the direct effect of VEGF on different types of immune cells is illustrated. Consequently, anti-VEGF (bevacizumab) can counteract these changes and therefore potentiate the efficacy of immunotherapy (atezolizumab) as we will describe here [40,106,107]. As the liver is an immunotolerant organ that deals with the continuous stream of antigens from the gut and HCC usually originates in a context of chronic liver inflammation leading to immune exhaustion, cancer cells in HCC benefit an immunotolerant TME [106]. This immunosuppressive nature of HCC is highlighted by KEYNOTE-240 and Checkmate 459, two phase 3 trials using ICI monotherapy in unresectable HCC, where significance for its primary outcome of OS could not be achieved [13,14]. An immunosuppressed TME is strongly associated with repressed anticancer immunity which impacts the effectiveness of immunotherapy. The goal of combining VEGF-inhibition with PD-L1-inhibition is to **enhance that anticancer immunity by changing the immunosuppressed TME to a more immunofavorable TME** [107]. Cancer immunity was characterized by Chen and Mellman as a seven-step, self-propagating, cyclical process referred to as the cancer-immunity cycle (CIC). It consists of three phases: (1) recruitment and activation of immune effector cells (steps 1–3); (2) trafficking and infiltration of T-cells into tumors (steps 4–5) and (3) recognition and killing of cancer cells (steps 6–7) [108]. In Figure 2, the CIC is illustrated and the mode of action of atezolizumab and bevacizumab upon it is added.

#### 5.1.1. Upregulation of Antigen Presentation Via Dendritic Cell (DC) Maturation and Functioning (CIC Steps 1–3)

T-cell priming and activation of CTLs is dependent on antigen presentation to T-cells in the lymph node for which DCs play a key role [108]. Tumor-associated DCs exist in an immature state and need to undergo maturation before becoming functional. This maturation, which is characterized by upregulating MHC I and II and other costimulatory molecules, is under the regulation of NF-ĸB. VEGF can, via VEGFR1, inhibit the NF-ĸB-pathway resulting in an impairment of DC maturation and consequently of the amount of mature DCs [109]. Bevacizumab treatment has been shown to increase the number of mature DCs in the peripheral blood of cancer patients [110]. Furthermore, DCs are also regulated by PD-L1 and as VEGF can upregulate PD-1 and PD-L1 on DCs, it results in suppressed functioning. On the other hand, anti-PD-L1 therapy can improve DC function and augment T-cell priming [111]. 

Taken together, both VEGF and PD-L1 have regulatory properties on DCs. Anti-VEGF antibodies, including bevacizumab, augment the maturation, and functioning of DCs, leading to an upregulated antigen presentation and priming and activation of T-cells restoring the anti-cancer immunity.

#### 5.1.2. Upregulation of T-Cell Proliferation, Trafficking, and Infiltration (CIC Steps 4–5)

To attack cancer cells, primed CTLs must move from the lymph node to the TME, entering the tumor vasculature, attaching to the endothelium, and migrating across the wall [108]. Due to the leakiness and chaotic structure of the tumor vasculature, it is more difficult for T-cells to enter [106]. VEGF also impairs T-cell adhesion by attenuating the expression of adhesion molecules (e.g., intracellular adhesion molecule 1 (ICAM1) and vascular cell adhesion molecule 1 (VCAM1)) on the vascular endothelium of the tumor and immune cells and it can upregulate clustering of abnormal adhesion molecules, which together leads to a reduced T-cell adhesion [112]. Furthermore, VEGF-A cooperates by inducing the Fas ligand antigen on endothelial cells that acquire the ability to induce apoptosis of CTLs but not Tregs [113]. Finally, VEGF can, by upregulating PD-(L)1 result in T-cell exhaustion [114]. Theoretically, anti-VEGF therapy prevents all of these actions leading to an improved T-cell flux to the tumor vasculature, better adhesion and migration through the endothelium, and better CTLs [72].

#### 5.1.3. Impairing Recruitment and Proliferation of Immunosuppressive Cells (CIC Steps 6–7)

VEGF can augment a set of immunosuppressive cells in the TME. Myeloid-derived suppressor cells (MDSCs), a heterogeneous population of cells constituting granulocytic and monocytic subsets that is upregulated by VEGF suppress the proliferation of CTLs and promote Treg development [115]. Moreover, immunosuppressor cells, such as MDSCs, can also drive angiogenesis, thereby creating a vicious cycle of immunosuppression. Moreover, the recruitment and proliferation of Tregs are mediated by VEGF directly. Finally, VEGF promotes M2-like tumor-associated macrophages (TAMs; prominent component of the leukocyte population of solid tumors, which displays an ambivalent relationship with tumors depicting an anti-tumor M1-phenotype and a pro-tumor M2-phenotype) in favor of the M1 phenotype [40]. Therefore, anti-VEGF will shift an immunosilenced TME with MDSCs and Treg toward a more immunopotent TME with a higher M1:M2 ratio and CTLs.

### 5.2. Clinical Efficacy of Atezolizumab with Bevacizumab

The first clinical trial consolidating the extensive preclinical evidence of the synergistic and complementary effect of the combination of anti-VEGF with ICI in aHCC was GO30140. In arm F of this open-label phase 1b study, 119 patients with untreated aHCC were randomly assigned to atezolizumab/bevacizumab or atezolizumab monotherapy. The side-effect profile of the combination was acceptable and the primary endpoint of PFS was significantly better for atezolizumab/bevacizumab versus atezolizumab (5.6 months versus 3.4 months (HR: 0.55 [0.40–0.74], *p* = 0.01)) [116]. These results were subsequently confirmed in a large registrational trial. IMbrave150 was conducted as a global, multicenter, open-label, phase 3 RCT to determine the safety and efficacy of atezolizumab/bevacizumab versus sorafenib in the first-line treatment of aHCC with coprimary endpoints of OS and PFS [15].

Between March 2018 and January 2019, 501 patients were randomly assigned in a 2:1 ratio to receive atezolizumab/bevacizumab (336 patients) or sorafenib (165 patients). Baseline characteristics (e.g., age, Child–Pugh (CP) score, BCLC-stage, macrovascular invasion, AFP) were generally well balanced between the two groups. The main inclusion criteria were >18 years, locally advanced, metastatic or unresectable disease, no previous systemic treatment for HCC, ECOG PS 0 or 1, CP A liver function, and adequate hematologic and organ function. The main exclusion criteria were an autoimmune disease, coinfection with the hepatitis B or C virus, and untreated or incompletely treated esophageal or gastric varices. Coprimary endpoints were OS and PFS (according to RECIST 1.1). Secondary endpoints included ORR (complete or partial response), duration of response (both according to RECIST 1.1 and modified RECIST), time to deterioration of quality of life and physical functioning (according to the European Organization for Research and Treatment of Cancer (EORTC) quality-of-life-questionnaire for cancer EORTC QLQ-C30) and safety profile (according to the National Cancer Institute Common Terminology Criteria for Adverse Events version 4.0).

The updated efficacy and safety data from IMbrave150 empower the results of the primary analysis [117]. There was a median follow-up of 15.6 (95% CI 0–28.6) months overall: 17.6 (95% CI 0.1–28.6) months in the atezolizumab/bevacizumab arm and 10.4 (95% CI 0–27.9) months in the sorafenib-arm. Median OS was 19.2 months for atezolizumab/bevacizumab and 13.4 months for sorafenib (HR [95% CI]: 0.66 [0.53–0.85], *p* < 0.001). Median PFS was 6.9 months for atezolizumab/bevacizumab and 4.3 months for sorafenib (HR [95% CI]: 0.65 [0.53–0.81], *p* < 0.001). The OS benefit with atezolizumab/bevacizumab *versus* sorafenib was generally consistent across all patient subgroups, except in the subgroup of patients with HCC from non-viral etiology (HR for death 1.05; 95% CI 5.6–9.6). PFS and ORR were consistent in all subgroups. There has been an 18% (95% CI 11–26, *p* < 0.001) difference in ORR between both arms in favor of atezolizumab/bevacizumab. The median duration of confirmed response was 18.1 (95% CI 14.6-not reached) months with atezolizumab/bevacizumab and 14.9 (95% CI 4.9–17.0) months with sorafenib. The safety profile of combination therapy was considered acceptable. Treatment-related serious adverse events occurred in 23% in the atezolizumab/bevacizumab arm and in 16% in the sorafenib arm. The most common treatment-related adverse events with atezolizumab/bevacizumab were proteinuria (29%), hypertension (28%), and increased aspartate aminotransferase (16%), whereas it was palmar–plantar erythrodysesthesia syndrome (48%) and diarrhea (44%) in the sorafenib arm. The percentage of patients who needed to discontinue treatment because of adverse events was 22% in the group of combination therapy and 12% in sorafenib-arm. In the group of combination therapy, a more delayed deterioration of patient-reported quality of life (11.2 months versus 3.6 months (HR [95% CI]: 0.63 [0.46–0.85]) and of physical functioning (13.1 months versus 4.9 months (HR [95% CI]: 0.53 [0.39–0.73]) was observed compared to sorafenib-group.

### 5.3. Potential Biomarkers for Combination Therapy of Atezolizumab with Bevacizumab

A recent study integrated transcriptomic, genetic, and immune histochemistry data of 358 tumor biopsies of patients treated with atezolizumab and bevacizumab in the context of the GO30140 phase 1b and the IMbrave150 phase 3 trial, in order to identify potential biomarkers and mechanisms for the responses. Overall, the authors confirmed the predictive power of immune cell signatures to predict the outcome of aHCC patients treated with immunotherapy. Responders to atezolizumab/bevacizumab had higher expression of genes linked to the adaptive and innate immune system, as well as expression of CD274 (PD-L1 mRNA) and a signature of effector T-cells. Moreover, immunohistochemically, tumors of responders were characterized by higher CD8+ T-cell infiltration and a trend toward higher PD-L1 expression. Response to atezolizumab/bevacizumab relative to sorafenib was also higher in tumors expressing lower levels of GPC3 (Glypican-3) and AFP, which were CTNNB1 wild type and carried TERT (telomerase reverse transcriptase) mutations [118].

Because of the existence of an atezolizumab monotherapy arm in the GO30140 phase 1b trial, the authors could also explore the clinical (instead of preclinical) proof of evidence of the synergistic effect of bevacizumab added to atezolizumab. The findings were confirmed by in vivo efficacy in an immunogenic HCC mouse model. No individual genes or pathways associated with the response between the two treatment groups were identified. However, by deconvolution of gene expression profiles, immune subsets including CD8+ T-cells, Tregs, and macrophages, were associated with an added clinical benefit of bevacizumab. In general, this study withholds an increased number of mature DC and CD8+ T-cells and a decrease in MDSC and inhibition of proliferating Tregs as a result of adding bevacizumab; which is in line with the above-described preclinical-based mechanisms [118].

Several biomarker analyses of clinical trials in RCC can provide further insights into the mechanisms of IO-VEGF synergism. In a phase 1 trial, where patients were initially treated with bevacizumab followed by combination treatment with atezolizumab/bevacizumab, paired biopsies pre- and post-treatment revealed that bevacizumab alone induced favorable changes to the tumor vasculature, resulting in reduced neoangiogenesis and vascular density. After subsequential combination therapy, tumors had increased infiltration of CD8+ T-cells, upregulation of MCH I expression, T-helper (a subset of T-cells expressing CD4 on their surface stimulating the activity of other immune cells by releasing cytokines), and CD8+ T effector signatures [119]. A phase 2 trial compared atezolizumab/bevacizumab, atezolizumab monotherapy, and sunitinib (IMmotion150 trial). In an exploratory biomarker analysis, gene expression signatures were derived from transcriptomic data, reflecting pre-existing T effector signaling, immunosuppressive myeloid infiltration, and angiogenesis. In tumors with high expression of the T effector signature and low myeloid inflammation, atezolizumab monotherapy had comparable efficacy to atezolizumab/bevacizumab, but in tumors with high myeloid infiltration, the addition of bevacizumab improved outcomes. These results indicated that the addition of anti-VEGF therapy might aid to overcome myeloid-driven resistance to ICI [59]. A biomarker analysis of the subsequential phase 3 trial, which compared atezolizumab/bevacizumab with the VEGFR-TKI sunitinib (IMmotion151) identified seven distinct molecular subsets based on an unsupervised transcriptomic analysis. Subsets characterized by high angiogenesis can be effectively treated with both atezolizumab/bevacizumab and sunitinib, whereas tumors with high expression of effector T-cells and/or cell-cycle signatures achieve better response upon treatment with atezolizumab/bevacizumab [62].

**Table 1:** Candidate biomarkers in HCC and RCC. AFP: alpha-fetoprotein; VEGF-A: vascular endothelial growth factor-A; Ang2: angiopoietin-2; SNP: single nucleotide polymorphism; VEGFR: vascular endothelial growth factor receptor; KDR: kinase insert domain receptor; PD-(L): programmed death-(ligand); TPS: tumor proportion score; CPS: combined positivity score; CTNNB: catenin beta; TMB: tumor mutational burden; IFN-y: interferon-gamma; RNA: ribonucleic acid; TCR: T-cell receptor; CD: cluster of differentiation; CXCR: chemokine receptor; NLR: neutrophil-to-lymphocyte; GPC3: glypican-3.

Antiangiogenics		Immune Checkpoint Inhibitors		Atezolizumab/Bevacizumab	
HCC	RCC	HCC	RCC	HCC	RCC
AFP (in particular for ramucirumab; [16,39]	Soluble VEGF [50,51,52]	Expression of PD-L1 e.g., TPS, CPS [9,10]	NLR-ratio [96,97]	Immune cell signature (genes linked to the adaptive and innate immune system) corresponding to upregulated PD-L1 expression and effector T cells [116]	Gene expression signatures reflecting the high expression of effector T cells and high myeloid infiltration in tumor tissue [58]
Soluble VEGF-A [45]	SNP in VEGFR1 [53,54]	Downregulated Wnt/β-catenin signaling e.g., CTNNB1-wt [80,86]	Transcriptomic immune-related gene signatures [59]	High CD8+ T cell infiltration and PD-L1 expression on immunohistochemistry [116]	
Ang2 [47,48]	Transcriptomic angiogenesis-related gene signatures including e.g., *KDR* [57,58,59]	High TMB [89,90]	Single-cell TCR-sequencing (in particular maintenance of TCR-clonality) [103]	Low levels of GPC3 and AFP [116]	
		IFN-y gene signaling [91]		CTNNB1-wt or TERT-mutation [116]	
		Single-cell TCR-sequencing: TCR-clonality and TCR-sharing between tumor and blood [92]			
		CXCR + CD8+ effector memory T cells in blood [93]			
		CD103+ tissue-resident memory T-cells [78]			

## 6. Conclusions and Further Directions

In recent years, we have seen dramatic progress in the outcomes of aHCC patients treated with systemic therapy. While trial-eligible patients historically had a mOS of up to one year, the most recent clinical trials reported about 20 months of OS with new immunotherapy combinations, such as atezolizumab/bevacizumab.

These advancements could be further refined by (1) thoroughly unraveling the mechanisms of action of new treatment(s) (combinations) and by (2) identifying markers that can predict the benefits. Both features, evidently, are intertwined, as an optimal biomarker ideally represents certain key features of the working mechanism. Combination treatments are challenging in this regard, as it is difficult to separate the effects of two treatments most often administered together. Moreover, the theoretical synergy of a combination treatment, supported by preclinical evidence, will not always be confirmed in the setting of a clinical trial [120].

In this review, we summarized the current state-of-the-art in terms of the mode of action and biomarker development of antiangiogenic agents, ICI, and the atezolizumab/bevacizumab combination. Comparing two cancer types with very similar drug class activities, HCC and RCC, reveals more similarities than differences. The findings described above might help in future clinical and preclinical research.

To make further progress in this area, new clinical trials must be maximally enriched with biomarker research. Certainly, double-armed trials are interesting because they allow the possibility to differentiate prognostic from predictive value. Nevertheless, candidate predictive biomarkers must, in the end, be prospectively validated (e.g., REACH-2 trial).

Ultimately, this knowledge should contribute to increased personalization of first-line systemic treatment in aHCC, as well as informed sequencing of subsequent lines.
